# Annotations

**Published:** 1910-05-21

**Authors:** 


### ANNOTATIONS.

### Miss Florence Nightingale.

Medical men as well as nurses will cordially congratulate Miss Florence Nightingale, who last week celebrated the 90th anniversary of her birthday. It is an insult to the profession to suppose that there is a member of it ignorant of this venerable lady's name, or who need be reminded of the good work that she has done, and still, by her unfailing interest in nursing, is doing. As medical men we are more able to appreciate the moral and practical influence that her actions have had than the public can. They have quickened and stimulated the medical no less than they have woke up the nursing profession. One of the glories of English hospitals, of which they may justly feel proud, is the fact that their nursing staffs are second to none anywhere in the world. Without that pre-eminence in nursing much of the pioneering work that is being done in hospitals would be impossible, or, at least, would have to be carried on under difficulties the severity of which only those who are cognisant of the state of affairs that prevailed in London institutions in the early fifties will be able fully to realise. The development of nursing under existing conditions, the betterment of the nursing profession as a whole, and the establishment of many aids to nursing in the shape of helping funds and training schools—all these, it is no gross exaggeration to say, we owe largely to the initiative and energy of Miss Nightingale. Her great services, not merely to her own profession, not merely to English nursing and English medicine, but to humanity at large, have been recognised, though it is legitimate to doubt if they can ever be adequately rewarded by any honours that the nation or the profession is able to bestow. The late King created her a member of the Order of Merit, and had she wished Miss Nightingale could have had other, though scarcely greater, distinctions conferred upon her. But it has always been her desire to do good unostentatiously and in the background, and she has shrunk, almost pathetically when one considers her great age and the fact that her health is no longer unbroken, from publicity. Nevertheless, on this occasion she will hardly take it amiss that the nation comes to her door with the expression of its sincere congratulation, and couples with that its grateful thanks for what she has done on its behalf.

### The late Dr. Gordon-Stables.

On Tuesday, May 10, at The Jungle, Twyford, Berks, died William Gordon-Stables, in his 71st year. Dr. Gordon-Stables, who was better known to the public as a novelist and writer of "juvenile fiction" than as a member of the medical profession, was an old Royal Naval surgeon, who graduated M.D., C.M., at Aberdeen in 1862, and retired from practice many years ago. As a surgeon in the Navy he saw service in many quarters of the globe and travelled extensively. He contributed largely to the lay Press, his articles being always well written and thoughtful, though it is possible that "trade-unionists" in the profession may have deemed him too eager to initiate the public into the sacred and untouchable mysteries of medicine. But Gordon-Stables would have been the last man to have cared a rap about the censure of a divisional meeting. For him medical etiquette was summed up in the sentence "Do to others as you would be done by." He saw no sin in putting his name to a column of "popular medical notes" in a daily paper, and many of the articles he contributed in this way are excellent educative *résumés*. He had a fluent, facile style and a fine sense of humour which stood him in good stead on many occasions. His love for an outdoor life was almost a passion, and during the last twenty years of his busy and strenuous career he found relaxation in travelling about in his caravan, appropriately styled "The Wanderer." His death removes one of the most popular of boys' novelists, and will be sincerely mourned by many thousands who have derived profit and pleasure from his tales of adventure and heroism.

### Output of Drugs and Chemicals in the United Kingdom.

Some interesting particulars concerning the value of the drugs and chemicals manufactured in the United Kingdom are contained in a report which has just been issued by the Board of Trade, compiled from the returns received under the Census of Production Act. This is the first time that reliable figures relating to the value of the drug and chemical manufacturers of this country have been available. Forms were sent to all manufacturers engaged in this industry, on which they were compelled by the Act to state the articles manufactured, the quantities manufactured, the quantity sold, the value of the articles, the number of workpeople employed, and so on, the object being to ascertain the total production of the United Kingdom during a period of twelve months. The gross output of factories engaged in the manufacture of chemicals, coal-tar products, drugs, and perfumery is given as £23,447,000, and the cost of materials used as £13,974,000. The number of persons employed is 51,088. On account of the varied nature of the products of these industries, many of which pass through several different branches and have been recorded at each stage by the different manufacturers, there is no doubt that this figure (£23,447,000) contains a large amount of duplication, and it is difficult to frame a close estimate of the value of the products of the industry as a whole. For instance, acids, essential oils, etc., are raw materials for the manufacture of other chemical products, but are also largely sold to firms outside the chemical industry. The following are the values of a few of the drug and chemical products manufactured during the period of twelve months: Drugs and galenical preparations, £2,461,000; fine pharmaceutical chemicals (including alkaloids, chloroform, ether, etc.), £1,613,000; patent medicines, £1,285,000; prepared food for infants and invalids, £571,000; druggists' sundries, £1,280,000; essential oils, £91,000; perfumed spirits, £302,000.

